# Basal Ganglia Calcification Secondary to Postsurgical Hypoparathyroidism Presenting With Seizures and Acute Confusion: A Case Report (Fahr Syndrome)

**DOI:** 10.7759/cureus.100553

**Published:** 2026-01-01

**Authors:** Fahad S Alrashidi, Ashwag H Mohajab

**Affiliations:** 1 Department of Internal Medicine and Nephrology, Ad Diriyah Hospital, Riyadh Third Health Cluster, Riyadh, SAU; 2 Department of Internal Medicine, Ad Diriyah Hospital, Riyadh Third Health Cluster, Riyadh, SAU

**Keywords:** endocrine disorders, fahr’s syndrome hypoparathyroidism, nephro critical care, neuro-critical care, neuro radiology

## Abstract

Fahr syndrome is a rare neurologic condition characterized by bilateral intracranial calcifications, most commonly associated with metabolic or endocrine disorders, particularly hypoparathyroidism. We report a middle-aged woman with post-surgical hypoparathyroidism who presented with acute confusion and a generalized tonic-clonic seizure. Laboratory evaluation revealed severe hypocalcemia with undetectable parathyroid hormone levels. Non-contrast computed tomography (CT) of the head demonstrated extensive, symmetrical calcifications predominantly involving the basal ganglia, with additional thalamic and scattered subcortical involvement, consistent with Fahr syndrome. The patient was treated with intravenous calcium followed by oral calcium and vitamin D supplementation, with gradual neurologic improvement. This case emphasizes that basal ganglia calcifications in hypoparathyroidism are often an incidental radiologic finding and underscores the importance of early, sustained metabolic correction to prevent recurrent hypocalcemic complications and potential clinical progression.

## Introduction

Basal ganglia calcifications are radiologic findings with a broad differential diagnosis ranging from incidental age-related changes to clinically significant neurologic disease. The term primary familial brain calcification (PFBC) (historically referred to as *Fahr disease*) describes progressive, typically bilateral calcifications associated with genetic variants and variable neurologic or psychiatric manifestations. In contrast, secondary basal ganglia calcification (*Fahr syndrome*) occurs in association with an identifiable underlying condition, most commonly disorders of calcium-phosphate metabolism such as hypoparathyroidism.

Distinguishing PFBC from secondary etiologies is clinically important because management priorities differ: PFBC prompts consideration of genetic counseling and family evaluation, whereas secondary calcifications require identification and correction of a reversible metabolic driver to prevent recurrent symptoms and further complications. Hypoparathyroidism, particularly post-thyroidectomy, can lead to persistent hypocalcemia and hyperphosphatemia and may be complicated by seizures, altered mental status, and intracranial calcifications.

We report a case of extensive symmetric intracranial calcifications in a patient with postsurgical hypoparathyroidism who presented with acute confusion and a generalized tonic-clonic seizure, highlighting a practical diagnostic approach and the importance of addressing adherence and long-term biochemical monitoring.

## Case presentation

A middle-aged woman with a history of hypothyroidism and postsurgical hypoparathyroidism, with irregular adherence to calcium and vitamin D supplementation, presented with acute confusion and a witnessed generalized tonic-clonic seizure. Non-adherence was attributed to a misunderstanding of the chronic need for replacement therapy, intermittent refill/access issues, and gastrointestinal intolerance. She also reported fatigability and constipation, with no paresthesias, and denied prior episodes of severe symptomatic hypocalcemia.

She had undergone a total thyroidectomy in 2021 for compressive symptoms, after which she developed hypoparathyroidism. There was no family history of similar neurologic disease or intracranial calcifications. She denied recent trauma, infectious symptoms, or toxin exposure.

On examination, she was confused but hemodynamically stable. Neurologic assessment revealed impaired consciousness (Glasgow Coma Scale 13/15) without focal neurologic deficits. There was no evidence of neuromuscular irritability, as indicated by negative Chvostek and Trousseau signs.

Laboratory evaluation demonstrated severe hypocalcemia (corrected calcium 1.8 mmol/L) with undetectable parathyroid hormone (PTH). Phosphate was elevated (2.5 mmol/L), 25-hydroxyvitamin D was low (20 ng/mL), and magnesium was mildly low (0.6 mmol/L). Additional abnormalities included macrocytic anemia and thrombocytopenia, with hematology work-up ongoing (Table 1).

**Table 1:** Key laboratory results on presentation, including electrolytes, renal function, and mineral-bone parameters. WBC, white blood cell count; 25-OH vitamin D, 25-hydroxyvitamin D

Test	Result	Reference range
Potassium	2.8	3.5-5.0 mmol/L
Sodium	139	135-145 mmol/L
Magnesium	0.6	0.7-1.0 mmol/L
Creatinine	95	60-110 µmol/L
Bicarbonate	26	22-28 mmol/L
WBC	7.8	4-11 × 10⁹/L
Corrected calcium	1.8	2.2-2.6 mmol/L
Phosphate	2.5	0.8-1.5 mmol/L
Albumin	35	35-50 g/L
25-OH vitamin D	20	30-100 ng/mL

Electrocardiography (ECG) showed a normal sinus rhythm with a QTc of 330 ms, without QT prolongation.

**Figure 1:**
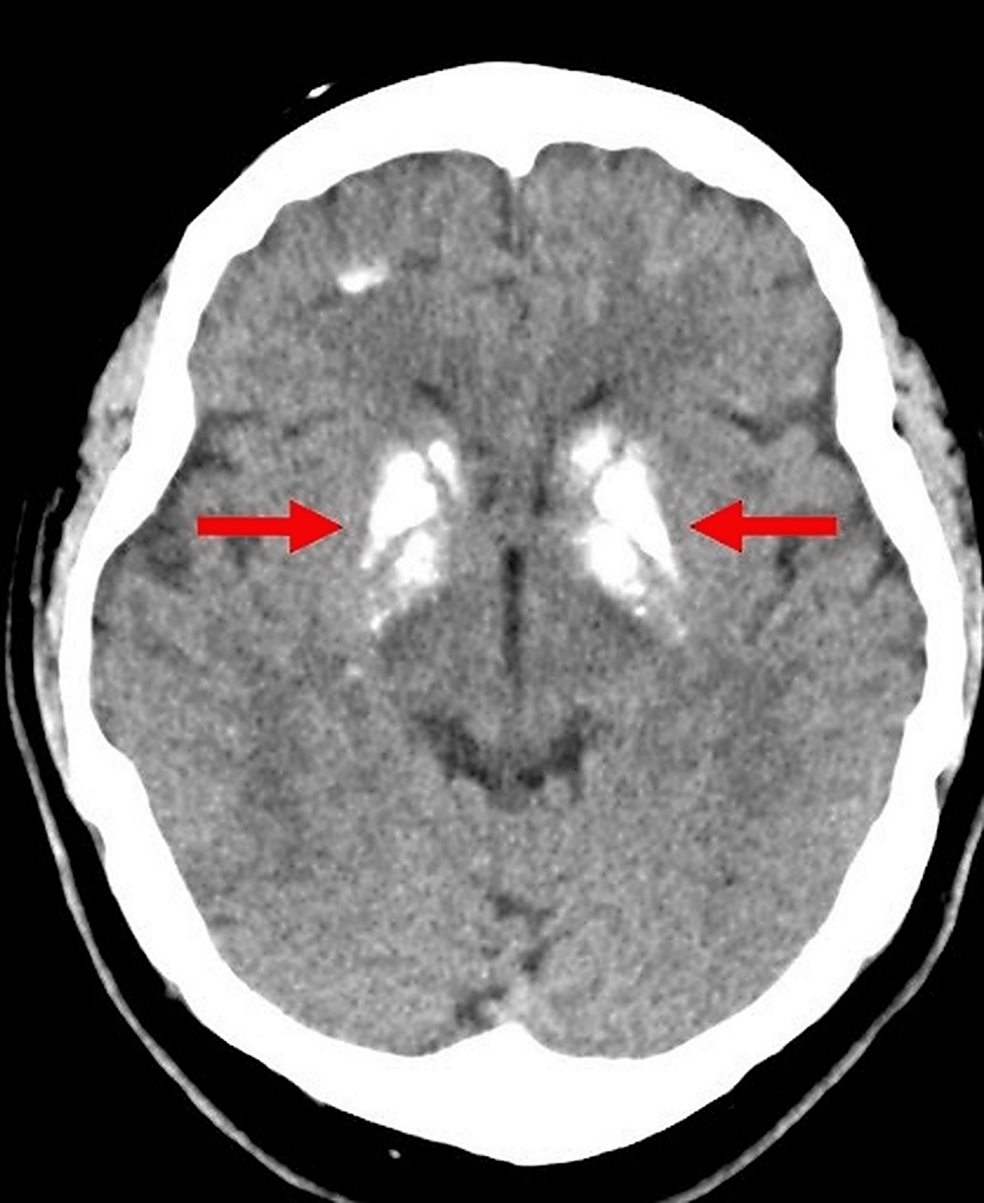
Non-contrast computed tomography (CT) scan of the head demonstrating extensive, bilateral, symmetrical intracranial calcifications predominantly involving the basal ganglia and thalami, with additional scattered subcortical calcific foci. Arrows indicate representative calcifications, consistent with Fahr syndrome in the setting of hypoparathyroidism.

A non-contrast computed tomography (CT) scan of the head demonstrated extensive, symmetrical intracranial calcifications, predominantly involving the basal ganglia and thalami, with additional scattered subcortical calcified foci (Figure 1). Arrows indicate representative areas of calcification.

Differential diagnosis

PFBC was considered in view of the extensive, symmetrical intracranial calcifications. However, the presence of a clear secondary metabolic driver - undetectable parathyroid hormone in the setting of severe hypocalcemia and hyperphosphatemia - together with the post-thyroidectomy clinical context and absence of a family history of similar neurologic disease or intracranial calcifications, strongly supported a secondary etiology (hypoparathyroidism-related calcification). Therefore, genetic testing for PFBC was not pursued.

Treatment

The patient was treated acutely with intravenous calcium gluconate, followed by transition to oral calcium and active vitamin D replacement. Levetiracetam was initiated at a low dose for seizure management. Electrolytes were monitored closely with serial calcium/phosphate measurements, and contributory abnormalities (including mild hypomagnesemia) were addressed as part of metabolic optimization. Endocrinology guided longer-term replacement therapy and biochemical monitoring.

Given the marked hyperphosphatemia, a phosphate binder was prescribed (sevelamer 800 mg three times daily with meals).

Outcome and follow-up

Her neurologic status improved gradually during hospitalization with correction of the metabolic derangements. She was discharged with outpatient endocrinology follow-up on calcium carbonate 1,200 mg three times daily between meals, sevelamer 800 mg three times daily with meals, and alfacalcidol (One-Alpha) 1 mcg daily. She received ongoing counseling regarding strict adherence, given prior irregular use related to a misunderstanding of chronic need, refill/access issues, and gastrointestinal intolerance, and was advised to continue regular monitoring of calcium and phosphate levels.

## Discussion

Fahr syndrome was originally described by Karl Theodor Fahr in 1930 and refers to secondary basal ganglia calcification, whereas PFBC describes genetically mediated intracranial calcification syndromes with overlapping neuroimaging phenotypes [1-4]. In clinical practice, secondary etiologies are frequently related to hypoparathyroidism and chronic disturbances in calcium-phosphate metabolism [3,5-7]. Sustained hypocalcemia with hyperphosphatemia is believed to promote calcium-phosphate deposition within the basal ganglia and other vulnerable regions, producing the characteristic pattern of bilateral, symmetric intracranial calcifications [2,3,6].

Neuroimaging is central to diagnosis. Non-contrast CT is more sensitive than MRI for detecting and characterizing calcifications and is therefore the preferred modality when Fahr syndrome is suspected [4,6,7]. The clinical spectrum is heterogeneous, ranging from incidental findings to seizures, movement disorders, psychiatric manifestations, and cognitive impairment [4,6]. In the present case, the acute presentation (generalized seizure and encephalopathy) was most plausibly precipitated by severe hypocalcemia, while the intracranial calcifications were likely chronic and incidentally identified during evaluation of the acute event. This distinction is clinically important because CT findings themselves rarely direct acute neurologic management; instead, they should trigger a structured metabolic evaluation to identify reversible causes and prevent recurrence.

From a practical standpoint, symmetrical intracranial calcifications should prompt confirmation on non-contrast CT followed by focused laboratory assessment of the calcium-phosphate-PTH axis (including magnesium and 25-hydroxyvitamin D) before labeling the condition idiopathic [8-10]. Differentiating secondary Fahr syndrome from PFBC is important because management and counseling differ: secondary forms require correction of the underlying metabolic derangement, whereas PFBC management is primarily symptomatic with consideration of family counseling and genetic testing [6,7,11]. In this patient, undetectable PTH with severe hypocalcemia and hyperphosphatemia, together with the absence of a family history, supported a secondary etiology; therefore, genetic testing was not pursued (Table 2).

**Table 2:** Key distinguishing features between primary familial brain calcification (PFBC) and secondary basal ganglia calcification (Fahr syndrome). Credits: Created by the authors based on the cited literature [6,7]. PFBC, primary familial brain calcification; PTH, parathyroid hormone; Ca, calcium; PO₄, phosphate; 25(OH)D, 25-hydroxyvitamin D; CT, computed tomography; MRI, magnetic resonance imaging

Feature	PFBC (genetic; *primary*)	Secondary Fahr (e.g., hypoparathyroidism)
Primary driver	Genetic disorders of phosphate handling / neurovascular unit	Disturbed Ca-P-PTH homeostasis; systemic/toxic/infectious causes
Common genes	SLC20A2, PDGFRB, PDGFB, XPR1, MYORG, JAM2	Not gene-driven; depends on underlying cause
Typical clues	Often family history, progressive movement/neuropsychiatric symptoms	Hypocalcemic symptoms; endocrine/metabolic context
Age at presentation	Often 30-50 years (variable penetrance)	Any age (depends on etiology)
Imaging pattern	Bilateral symmetric basal ganglia ± dentate/thalamic/subcortical	Overlaps with PFBC; can be extensive with long-standing disease
Management emphasis	Symptomatic care; counseling/genetic considerations	Treat the cause (calcium/vitamin D ± calcitriol; phosphate control)

Long-term management of chronic hypoparathyroidism aims to maintain serum calcium in the low-normal range using calcium supplementation with vitamin D and/or calcitriol, while monitoring phosphate levels and urinary calcium excretion to reduce complications such as hypercalciuria and nephrolithiasis [8-10,12]. In addition, concurrent electrolyte abnormalities (e.g., hypomagnesemia and hypokalemia) may further lower seizure threshold; therefore, comprehensive electrolyte correction and cardiac monitoring (including QTc assessment) are integral to acute care. Finally, this case underscores a preventable pathway to recurrent hypocalcemic emergencies - irregular adherence to replacement therapy - highlighting the importance of structured follow-up and adherence counseling to reduce future events and potentially mitigate clinical progression [2,8,10,12].

## Conclusions

This case illustrates Fahr syndrome as a radiologic manifestation of postsurgical hypoparathyroidism, identified during evaluation of acute confusion and a generalized tonic-clonic seizure. Severe hypocalcemia with undetectable parathyroid hormone and hyperphosphatemia supported a secondary etiology, emphasizing the importance of promptly assessing the calcium-phosphate-PTH axis when bilateral, symmetrical intracranial calcifications are detected. Non-contrast CT remains the preferred modality for confirming intracranial calcifications. Management should prioritize sustained metabolic correction with calcium and vitamin D/calcitriol and structured follow-up to prevent recurrent hypocalcemic complications and potential clinical progression.
